# Approaches in metabolomics for regulatory toxicology applications

**DOI:** 10.1039/d0an02212h

**Published:** 2021-02-09

**Authors:** Eulalia Olesti, Víctor González-Ruiz, Martin F. Wilks, Julien Boccard, Serge Rudaz

**Affiliations:** a School of Pharmaceutical Sciences, University of Geneva Switzerland serge.rudaz@unige.ch; b Institute of Pharmaceutical Sciences of Western Switzerland, University of Geneva Switzerland; c Swiss Centre for Applied Human Toxicology (SCAHT) Switzerland; d Department of Pharmaceutical Sciences, University of Basel Switzerland

## Abstract

Innovative methodological approaches are needed to conduct human health and environmental risk assessments on a growing number of marketed chemicals. Metabolomics is progressively proving its value as an efficient strategy to perform toxicological evaluations of new and existing substances, and it will likely become a key tool to accelerate chemical risk assessments. However, additional guidance with widely accepted and harmonized procedures is needed before metabolomics can be routinely incorporated in decision-making for regulatory purposes. The aim of this review is to provide an overview of metabolomic strategies that have been successfully employed in toxicity assessment as well as the most promising workflows in a regulatory context. First, we provide a general view of the different steps of regulatory toxicology-oriented metabolomics. Emphasis is put on three key elements: robustness of experimental design, choice of analytical platform, and use of adapted data treatment tools. Then, examples in which metabolomics supported regulatory toxicology outputs in different scenarios are reviewed, including chemical grouping, elucidation of mechanisms of toxicity, and determination of points of departure. The overall intention is to provide insights into why and how to plan and conduct metabolomic studies for regulatory toxicology purposes.

## Abstract 2

This tutorial review provides an overview of key concepts in metabolomics and their application to develop successful strategies in regulatory toxicology-oriented studies.

## Graphical abstract

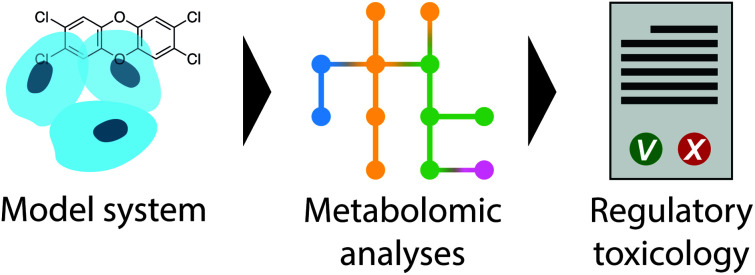

## 1. Introduction

To ensure the safe use of new substances or new uses of already existing substances, an assessment of toxicity hazards and risks is needed.^1,2^ Although toxicological evaluations of chemicals have continuously evolved, an increasingly complex chemical universe and novel toxicological problems such as endocrine disruptors have challenged the conventional procedures of chemical safety evaluation.^3–5^ In the current scenario of increasing demand, there is a need for new methodologies that are faster, cheaper, and less dependent on animals.^6,7^ The low throughput and elevated cost of traditional toxicity testing approaches render them impractical for assessing large numbers of chemicals.^8^ Against this background, the development and application of new methodologies in regulatory toxicology has begun during the past decade.^1,3^ The goal of these new approaches is to provide extensive and meaningful data to fill the existing information gaps (both hazard and exposure) using a weight of evidence (WoE) approach to ensure compound safety^1^ and reducing animal testing, cost and time. The use of *omics* approaches, especially metabolomics, has been discussed as a novel tool for regulatory toxicology and risk assessment.^1,2,9–11^

The term metabolomics refers to the measurement of small molecules that are part of the metabolism of a biological system (cell, tissue, organism, *etc*.) at a specific time point. These molecules have a low molecular weight (typically <1000 Da) and include compounds such as sugars, amino acids, fatty acids, nucleosides, and organic acids.^12,13^ Such metabolites are not only downstream products of genes, mRNA and proteins, but they also have the capacity to back-regulate the expression and activity of other biomolecules.^14^ The comprehensive and simultaneous analysis of hundreds of endogenous metabolites in a biological sample provides a direct snapshot of the biological activity in the studied system.^15^ Metabolomics is now widely established as a tool serving diverse applications in systems biology, such as diagnosing diseases, associating risk factors with pathologies, monitoring the effectiveness of a treatment or facilitating drug discovery and development.^16–18^ Metabolomics has also been applied in precision medicine^19^ and clinical pharmacology^20^ or even to discriminate potential positive cases of drug consumption^21^ among many other applications.^19,22^

In the early 2000s, metabolomics was proposed as a new technique to study drug toxicity.^23^ Shortly after, the Consortium for Metabolomic Toxicology (COMET)^24^ was created with the objective of predicting liver and kidney toxicity after exposure to several compounds through metabolomics.^25^ Since then, the use of metabolomics for toxicological purposes has increased exponentially.^9^ During recent decades, the implications of metabolic profiling in toxicological studies have moved toxicological evaluation from a rather descriptive strategy based on the observation of apical endpoints in experimental animals to a more targeted and mechanistic understanding of the effect of an initiating event on living systems.^9^

In the past few years, metabolomics has also found its place in the field of regulatory toxicology as a novel tool to improve the safety profile assessment of new compounds.^2^ Indeed, several panels of experts (gathering stakeholders from academia, industry and public agencies/government) are currently exploring the best uses and practices for metabolomics in chemical safety evaluation.^26^ While major advances have been made during the last decade, validating the use of metabolomic data for regulatory purposes remains an on-going goal.^1^ The main progress needed to fully adopt metabolomics in regulatory toxicology has been developing and widely adopting best practice guidelines to conduct metabolomic studies with regulatory purposes^26^ and performing relevant metabolomic studies addressing regulatory problems to showcase their potential.^27^

In this review, we aim to provide an overview of the metabolomic workflow for regulatory toxicology purposes through the study of the different experimental design strategies, the choice of an adequate analytical platform with a focus on separation techniques coupled to mass spectrometry (MS), and the adaptation of data treatment tools. Finally, this work presents illustrative recent cases where metabolomics has been shown to be a useful new element of the available toolbox for regulatory toxicology.

## 2. Metabolomic experiments for toxicology evaluation

**Fig. 1:**
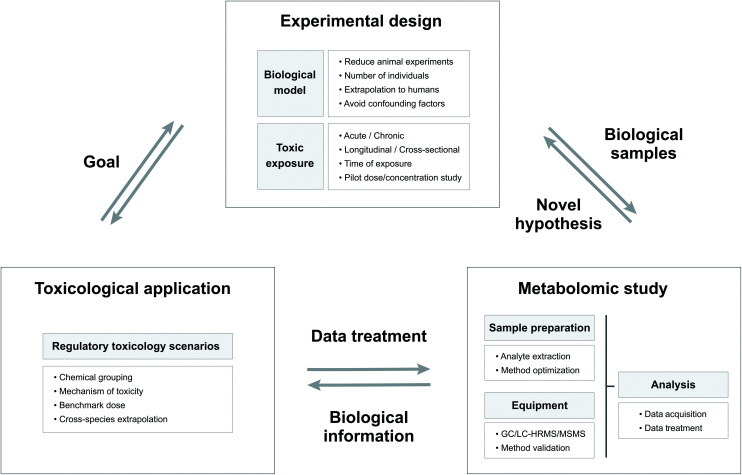
Scheme showing the relations between the experimental design, the metabolomic study and the toxicological applications with their principal characteristics.

The goal of toxicity evaluation is to generate information that identifies and characterizes hazards which, together with estimates of exposure levels, enables risk management to develop and implement measures that ensure adequate protection of public health.^28^ The in-depth study of changes in the endogenous metabolite content resulting from exposure to a chemical can provide relevant information for regulatory toxicology.^28^ In the framework of regulatory purposes, Fig. 1 shows a schematic representation of the links between the structure of the experimental designs, the nature of the metabolomic studies and the toxicological application.^26^

## 3. Experimental design

The experimental design should ensure that the biological samples collected will allow for the generation of quality analytical data to answer the experimental question through reliable data analysis,^29^ which requires careful planning to avoid biased results or a misinterpretation thereof.^30^ Complex questions or confounding factors can lead to challenging experimental designs involving several concurrent variables (*e.g.*, healthy population compared to different stages of diseases,^31^ different toxicants at several doses, *etc*.).^32^ During the setup of the experimental design, it is necessary to consider the inclusion of positive and negative controls (such as a vehicle group, a reference chemical, *etc*.) to ensure the relevance of the experiment and the reliability of the data.^30,32,33^ Overall, during the conception of the experimental design, several factors must be considered, mainly the toxicological exposure (parameters to monitor, for example, targeted endogenous metabolites) and the biological model (experimental design and sample preparation) along with other aspects such as the analytical platform to use (expected concentrations of the analytes and previous knowledge about them) and the data treatment, which are discussed in section 4.

### 3.1. Biological model

For decades, the toxicological evaluation of substances in the context of regulatory risk assessment has been mainly based on animal experimentation, typically aimed at finding no observable adverse effect level of the toxicant that could be extrapolated to humans.^34^ However, more recently great efforts have been made to investigate novel approaches with the aim of reducing or replacing animal testing.^3,35^ Many alternative animal use methods (such as 2D^36^ or 3D^37^ cell model systems) are being evaluated to ultimately serve the decision-making process towards the use of safe chemicals.^38^ In addition, there is an increasing focus on biomonitoring in human observational studies of environmental exposures as a source of biological samples to which metabolomics can be applied.^39^

Biological samples must contain the maximum possible amount of reliable, precise and accurate information while keeping the cost and animal usage to the minimum.^32^ Biological replicates must be carefully planned to provide an estimation of the biological and analytical variability while minimizing cost and invested resources.^29^ In metabolomics, some algorithms, such as MetSizeR^40^ or data-driven sample size determination,^41^ were developed for elaborating an optimized experimental design adaptable to any research project.^42,43^ In addition to the number of samples, the amount of biological material collected is also a crucial aspect that needs to be adjusted to the nature of the analysis and the goal of the study.^15^ Furthermore, choosing an adequate number of experimental factors to study is also highly dependent on the biological model and the toxicological goal.

Another key aspect that might affect the results obtained from an experimental setup is the possible presence of confounding factors, which are external parameters that may influence (in a direct or indirect manner) the outcome of the study and must be minimized and taken into account to prevent drawing incorrect conclusions. In cases where these factors cannot be controlled, they should be identified and considered during data treatment and biological interpretation. For example, in human studies demographic parameters (sex, age, ethnicity, geographical area, *etc.*), comorbidities, habits, diet, cycles, ambient exposures or medications may interfere and lead researchers to incorrect interpretations of the results.^43^ An example of a common confounding factor is the daily cycle of many metabolites (such as steroids) caused by circadian rhythms.^44–46^ For simpler biological systems such as cell cultures, growing conditions (*e.g.*, type of medium, temperature, environment, *etc*.) can be of key importance.^43^

### 3.2. Toxicant exposure

The experimental design of toxicological safety studies may range from simple case-controlled treatment experiments to more complex investigations that could involve multiple dose groups, different time points, several cohorts of animals and numerous experimental variables.^37^ Among the more common designs, there are crossover studies, factorial designs, or completely randomized experiments.^32,47^ Indeed, the selection of the monitored variables (such as the dose, the time of exposure, acute/chronic treatment, *etc*.) will be strongly linked to the goal of the study.

When exposing biological systems to a certain toxicant to evaluate its effects, attention must be paid to the applied concentration to trigger a measurable response without causing excessive cell death. In the case of cell cultures, conducting cell viability studies at different concentration levels before carrying out actual experiments is always a good practice. In addition, the concentrations potentially found in tissues taking into account realistic exposure scenarios and organism distribution properties should also be considered.

## 4. Metabolomic workflow for toxicology

In metabolomic workflows, the major steps implicated are (i) sample pretreatment and preparation, (ii) chemical analysis of metabolites, and (iii) suitable data treatment.

### 4.1. Biological sample collection, storage and preparation

By definition, metabolism is highly dynamic, and metabolites are constantly undergoing chemical reactions. Thus, the collection, pretreatment and preparation of biological samples is critical to obtain a representative snapshot of the system under study.^48^ Collection and storage of samples prior to sample preparation are key steps to ensure sample representativeness and stability.^49^ The use of standardized collection protocols and containers, the correct training of the staff in charge of sample collection, and the addition of preservatives can improve sample quality at these steps.^50^ As metabolism is inherently active, strategies such as immediate freezing after sample collection are required to avoid undesired metabolite degradation.^15^ Before measuring endogenous metabolites with the analytical platforms available (section 4.2), samples usually need to be submitted to cleanup procedures. Today, there is no general sample treatment that allows the measurement of the whole metabolome. Nevertheless, a number of generic sample preparation protocols have been widely adopted by the metabolomics community, as they have been proven to yield robust results and provide a good rugged starting point for further optimization.^51–53^ Depending on the type of chemical analyses to be performed, the nature of the metabolites to investigate and the type of samples, adequate sample preparation protocols must be applied to maximize metabolite recovery and minimize interferences. While highly concentrated analytes require a dilution step, low-concentration analytes demand a pre-concentration procedure to improve the sensitivity of the method.^15^ A number of sample preparation techniques involving different degrees of selectivity span from simple protein precipitation (PP) steps to solid-phase extraction (SPE) or liquid–liquid extraction (LLE).^54^ Some analytes require a chemical derivatization step to improve metabolite extraction, separation, ionization or detection.^55^ Moreover, it is key to use internal standards (generally ^2^H or ^13^C isotopically labelled analytes) to account for analyte losses, compound degradation, analytical performance of the different steps, and normalization issues.^56^ Overall, the diversity of sample matrices, chemical structures and challenges associated with analyte extraction must be comprehensively monitored to obtain accurate and reproducible results that could draw meaningful conclusions from the data.

### 4.2. Data acquisition

For a comprehensive measurement of the metabolic content of a sample, combinations of different techniques and acquisition strategies are commonly applied according to the nature of the study.

#### 4.2.1. Analytical platforms

Due to the broad variety of metabolite physicochemical properties and abundances, it is currently impossible to monitor the whole metabolome within a single analytical run.^57^ Properties such as polarity, stability or volatility challenge metabolomic analyses and require complementary and optimized analytical platforms for the detection, identification and quantification of endogenous metabolites.^58,59^ The improvement of instrument sensitivity and sample treatment and the combination of different analytical strategies become critical steps for greater metabolite coverage.^16^ The most widely used analytical tools in metabolomics analysis are nuclear magnetic resonance spectroscopy (NMR) and the combination of mass spectrometry (MS) with separation techniques,^60,61^ such as liquid chromatography (LC), gas chromatography (GC),^62,63^ supercritical fluid chromatography (SCF)^64,65^ or capillary zone electrophoresis (CZE).^66,67^ The choice of the separation technique is based on (i) the sample matrix, (ii) metabolite abundances, (iii) physicochemical properties of the metabolites and (iv) the amount of sample.^60^ In metabolomics, LC^15,68^ is one of the most commonly adopted separation techniques, and in the present review, we focus on this technique.

The choice of the stationary and mobile phases will determine which chemical properties of the compounds will drive the separation (*e.g.*, polarity, electrical charge, molecular size).^69,70^ To increase metabolome coverage, the combination of data acquired in several modes (such as reversed-phase LC –RPLC– for the separation of apolar compounds and hydrophilic interaction LC –HILIC– for polar metabolites) is commonly used.^32,71,72^ The combination of LC with MS detection provides a broad compound identification capacity with high sensitivity and robustness. MS is based on the formation of ions that are subsequently separated according to their mass-to-charge (*m*/*z*) ratio and eventually detected. MS instruments usually comprise the ionization source, a mass analyser and the detector. The outlet of the separation technique can be coupled to the MS instrument through different interfaces, allowing the ionization of a large range of chemical structures.^15^ Mass analysers are usually classified as either low or high mass resolution. The high-resolution analysers (such as time-of-flight (TOF) or orbital ion trap (Orbitrap)) have the ability to distinguish compounds with small mass differences, thereby enabling molecular formula determination from accurately measured mass (precision in the 0.1 mDa range or less). At the same time, they can be used to monitor a broad range of masses, which makes them the option of choice for so-called untargeted experiments, when a global overview of the chemical space is needed (see section 4.2.2). On the other hand, low-resolution mass analysers (such as the triple quadrupole (QqQ) or ion traps) are limited to the monitoring of a pre-defined set of masses. Nevertheless, they feature a better performance in terms of sensitivity and selectivity, which makes them extensively used for targeted metabolomics studies with quantitative purposes.^15^

#### 4.2.2. Acquisition strategy

For metabolomics studies, two main acquisition strategies are commonly used: untargeted and targeted approaches. The goal of untargeted acquisition is to provide unbiased and comprehensive monitoring of as many endogenous metabolites as possible in a biological sample without *a priori* knowledge. Its main challenge is the assignment of the acquired signals to putative or known metabolite identities, most often performed using databases and commercially available standards. It is commonly accepted that formal metabolite identification can be achieved when at least two orthogonal properties (such as chromatographic retention time, accurate *m*/*z*, mass fragmentation pattern and/or NMR chemical shift) match the experimentally measured properties of an authentic standard measured under the same conditions. Different levels of confidence have been defined by the metabolomics community depending on the kind of information used to annotate or identify each feature.^73,74^ Additionally, the multiplicity of adducts generated during the ionization process, as well as the presence of isobaric and co-eluting species, are known to be one of the main issues with annotation in ESI-based LC-MS untargeted metabolomics.^75^ The nature of the regulatory application will determine which minimum level of confidence is required in a particular toxicology metabolomic study.^76^

In the toxicology field, the untargeted metabolomics strategy has been shown to be a valuable hypothesis-generating tool.^77^ The monitoring of a large number of metabolites (on the order of thousands) in biological matrices after exposure to a toxic compound can provide a better understanding of the toxicant effect.^78^ In this manner, untargeted metabolomics can contribute to identifying specific mechanisms of toxic modes of action and markers of human chemical exposure.^59,79^ Another application of untargeted metabolomics in toxicology is its potential to determine the safety profile of drugs or pesticides under development. When applied to drug discovery, such toxicological screening aims to provide insights into the potential effects of new chemicals, and untargeted metabolomics can provide relevant information for accelerating toxicological evaluation.^2,23^ Furthermore, untargeted metabolomics could also provide insights into toxicological screenings with off-target drugs that cause adverse drug reactions.^80^

**Fig. 2:**
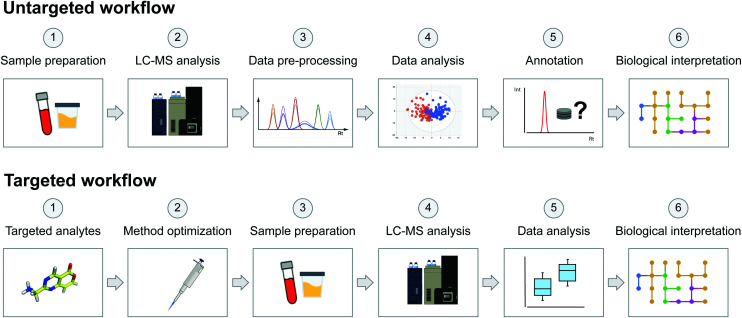
Schematic representation of untargeted and targeted acquisition workflows in metabolomics studies. The untargeted acquisition workflow was reproduced from Pezzatti *et al.*^43^ with permission from Elsevier, copyright [2019].

On the other hand, the targeted approach aims to analyse a predefined set of endogenous metabolites. These targeted metabolites are known, allowing the development and optimization of analytical strategies for the analysis of specific sets of molecules and metabolic pathways of interest.^12,13^ In general, targeted approaches focus on the quantification of a low number of metabolites, which can be crucial to determining physiological concentration ranges or reference clinical intervals, allowing us to distinguish normal variations from pathological or toxic levels.^56^ The main advantage of the targeted approach compared to the untargeted approach is an improved sensitivity, thus making it more appropriate for low-abundance species and quantitative experiments. In any case, the goal of the study will drive the choice of an untargeted or a targeted approach. In toxicological studies, targeted metabolomics can contribute to improving the understanding of toxicity pathways of hazardous chemicals^58,81^ by providing quantitative data useful for predicting the pharmacological profile of a studied substance.^82^ Targeted metabolomics also provides information for drug toxicity mechanism identification,^83^ toxico-kinetic monitoring^84^ and profiling xenobiotic bioactivation.^85^ The scheme in Fig. 2 highlights the main key differences between the two common acquisition workflows, from sample preparation to biological interpretation.

Both strategies (targeted and untargeted) are complementary and can be sequential. Thus, the analysis of a low number of samples with an untargeted acquisition generates data-driven hypotheses that then facilitate a closer monitorization of the relevant metabolites with a targeted methodology in larger sample sets.^86^

Finally, the combination of targeted and untargeted acquisition approaches in a single analysis (termed a “hybrid assay”) is also possible. It can rely on the acquisition of chemical information using an untargeted setup but with a special focus on a pre-selected set of metabolic compounds. A common approach is a sequence of two different acquisitions in which findings from a first untargeted measurement highlight a target set of compounds to be addressed in a second targeted experiment.

### 4.3. Data treatment

#### 4.3.1. Data handling/processing

Metabolomic data processing involves a series of procedures for extracting relevant biochemical information from a large number of measured signals before performing statistical analyses. The starting point of the workflow is called peak picking. This first step aims to convert raw data, *i.e.*, signal intensities recorded over mass and time dimensions into a series of ion features characterized by a peak area and a pair of *m*/*z* and retention time values. For that purpose, several algorithms are usually applied to extract reliable LC-MS information while removing irrelevant or noisy signals. After peak detection, multiple peaks corresponding to different isotopes, adducts and fragments of the same molecule can be grouped, thereby reducing chemical redundancy in the data.^87^ An alignment procedure is then usually applied to correct for retention time drifts between samples to assign the same feature in all observations to a given variable in the final data table.

Ion features are further filtered using parameters computed from quality control (QC) samples mandatory for any metabolomic experiments, which constitute repeated measurements of the same sample during the whole data acquisition process. QCs are usually injected at the beginning and end and in regular intervals during the sequence to monitor the stability of the measured signals. They can then serve as an objective basis for assessing the stability of the analytical process under real actual experimental conditions. Reliability indices such as relative standard deviation or response to dilution can be used to distinguish relevant peaks from noise.^71^ As previously mentioned, metabolite annotation is another important step and is usually carried out by comparing different properties of the ion features with reference values of metabolites stored in databases. These characteristics include accurate mass, fragmentation pattern, retention time, and collision cross-section. The use of one or several of these properties and the quality of their evaluation (in-house experimental data, databases or *in silico* predictions) will define the reliability of the annotation according to recognized reporting standards.^88^

Taken together, the different steps of the raw data processing workflow result in a data table where a collection of samples in rows (n observations) is characterized by the measured intensity of a series of metabolites in columns (p variables). For most toxicological studies using metabolomics, the size of the variable set is very often much greater than the number of samples, and dedicated data analysis strategies must be used to handle this type of structure. Both univariate and multivariate statistical techniques can be used to highlight the biochemical information hidden within the data.^89^ High-dimensional metabolic profiles can be efficiently handled using multivariate models to extract metabolic trends shared by subsets of compounds, while single-variable analysis is often very useful to assess the predictive merit of a properly identified metabolite. These two alternatives should therefore be seen as complementary tools.^90^

#### 4.3.2. Univariate statistical analysis

Single-variable statistical analysis using univariate techniques offers efficient solutions to detect biological modulations potentially due to toxic exposure by comparing metabolic levels between experimental conditions. The difference or ratio between central tendency parameters (mean or median) is classically used to investigate the statistical reliability of experimentally observed alterations. When a statistical test is carried out, a true difference due to an experimental factor can be distinguished from random variations between groups of observations. In the vast majority of cases, the results are expressed as a probability (*p*-value) and compared to the 0.05 (5%) threshold for statistical significance. This *p*-value is a widely adopted and simple measure (although very regularly called into question) that can be evaluated using either parametric techniques assuming specific distribution characteristics or their non-parametric counterparts.^91^ Standard tests comparing group means or medians included Student's test, one-way analysis of variance, the Wilcoxon rank-sum test and the Kruskal–Wallis test. Choosing an appropriate test depends on the distribution and scedasticity of the data as well as the potential dependence between observations. Because the risk of false positive results (type I error) increases when multiple hypothesis testing is carried out, an important precaution must be taken when a large number of metabolites are compared in parallel. In that case, dedicated methods accounting for the number of tests must be implemented to limit this effect by correcting the threshold applied to declare a difference as significant, *e.g.*, Bonferroni or Holm corrections, or by evaluating the false discovery rate, *e.g.*, using the Benjamini–Hochberg method.^92^

#### 4.3.3. Multivariate data modelling

Multivariate approaches are performed to extract patterns of variations associated with multiple variables to summarize a dataset and offer a condensed overview of complex high-dimensional metabolic profiles, highlighting subsets of potentially biologically relevant metabolites associated with specific trends such as the effects of toxic exposure.^93^ One of the methods of choice for the exploratory analysis of metabolomic data is principal component analysis (PCA). PCA makes use of covariances or correlations between variables, *i.e.*, redundancy of variability to build new synthetic dimensions in the data space. The scores correspond to the coordinates associated with the observations (samples) in the model. They therefore provide an efficient tool to detect subsets of samples sharing (at least partially) similar or different metabolic patterns (*e.g.*, for chemical grouping, see below). Moreover, this representation offers a potent way to highlight potential outlier observations characterized by an atypical metabolic profile. As a consequence, PCA is often implemented as the first step of the metabolomics data modelling workflow in toxicology. The coefficients of the measured variables, called the loadings, serve as an objective basis to evaluate the metabolic information. Both scores and loadings can easily be displayed as scatter plots and superimposed to interpret the PCA.

When the detection of subsets of observations sharing overall similar metabolic patterns is the aim of the study, clustering methods offer efficient strategies to detect natural partitioning in the data. For that purpose, distances between observations are computed by defining a function, *e.g.*, Euclidean and Manhattan distances, to define homogeneous subsets in the dataset, called the clusters. A large diversity of clustering techniques is available, although hierarchical cluster analysis (HCA) constitutes an interesting choice when the number of natural clusters is unknown. Moreover, the results of this method are easy to interpret as a tree graph, called the dendrogram, that defines the hierarchical structure of similarity among groups of observations, while the leaves of the tree correspond to a single sample. When more than one observation is included in a cluster, a linking function is defined to assess the distances between groups of observations. Cluster analysis is often implemented to treat either samples or metabolites independently, but co-clustering (or bi-clustering) constitutes an effective alternative to consider both rows and columns of the data table simultaneously.^94^

In many situations, variations of biological interest may be masked by greater sources of variation in the data, such as inter-individual differences. For example, known experimental groups of observations (*e.g.*, toxic exposure *vs.* control) may be difficult to distinguish based on unsupervised modelling. In this case, other methods taking additional information are mandatory. The goal of supervised approaches is to build models with the capacity to predict a response (Y vector or matrix) from the experimental data (X matrix). For that purpose, the model is trained to extract patterns in the independent variables X that are related to the dependent Y response. Partial least squares (PLS) regression is a very popular technique for building supervised models from metabolomics data.^95^ The interpretation of the PLS model is then comparable to PCA because the scores are the projections of the observations on the new axes, and loadings are the coefficients of the variables associated with the latent variables.^96^ Class membership can be used as a Y response when discriminant analysis (DA) is the aim of the study. In that case, PLS-DA models extract metabolic patterns, which maximize the separation between pre-specified experimental groups. In metabolomics, orthogonal PLS (OPLS) regression is a widely used extension of traditional PLS.^97^ The latter facilitates the interpretation of the model based on either predictive or orthogonal scores and loadings. Moreover, additional graphical outputs, such as S-plots or shared and unique structure (SUS) plots, were specifically developed,^98^ further increasing the popularity of this method in the metabolomics community.

Because *a priori* information is used in supervised learning, proper model evaluation is mandatory to ensure the reliability and stability of the models. Diagnostic parameters can be computed from the comparison of predicted and true response values obtained from resampling strategies such as cross-validation.^92^ However, it is not known which *R*^2^ or *Q*^2^ values truly correspond to a reliable model because it depends on the dimensionality of the dataset, *i.e.*, the number of samples and variables. In that context, permutation tests can help to evaluate the validity of the model. By analysing many versions of the data with randomly assigned responses (*e.g.*, class labels), a distribution following the *H*_0_ hypothesis is obtained. Each diagnostic parameter used to assess the quality of the model can then be compared to its *H*_0_ distribution to deduce from which value the model can be considered reliable. Alternatively, resampled observations with replacement, *i.e.*, bootstrap, can provide additional information on the stability of the model.^99^

## 5. Applications of metabolomics in toxicological regulatory sciences

The aim of regulatory toxicology is to prevent hazardous substances and products from producing adverse effects on human health and on the environment.^100^ In a recent work by Viant *et al.*, several experts in metabolomics and toxicology evaluated the main strengths of metabolomics for four toxicology regulatory scenarios.^11,26^ These scenarios include (i) chemical grouping, (ii) mechanisms of toxicity for understanding adverse outcome pathways (AOPs), (iii) discovering points of departure (PoDs) from benchmark dosing and (iv) for cross-species extrapolation of toxicity pathways.^26^

Although a limited number of examples addressing specific regulatory questions exist,^26^ many metabolomics studies have been developed with toxicological evaluation purposes. In the present review, we selected several relevant studies that were published to date and grouped them according to the most commonly found scenarios of application in regulatory toxicology.

### 5.1. Chemical grouping and read-across

Chemical grouping and read-across approaches take advantage of structural similarities between substances with known toxicology profiles and new chemical entities to infer the expected toxicological behaviour of the latter.^101^ This strategy is commonly used in regulatory toxicology as an alternative to animal testing,^35^ although in many cases, there is a lack of scientific evidence to support the prediction leads.^26,35^ This required scientific evidence could come from additional experimental data, such as the metabolism or the bioavailability of the studied compounds.^102^ Since the metabolome is one of the closest *omics* reflections of the phenotype, metabolic disturbance patterns appearing upon exposure of a test biological system to a new substance can be compared to those caused by a model compound, thus allowing the assessment of the similarities between both responses at the actual phenotypic level instead of just chemical structure similarities, which is much more relevant from a regulatory toxicology point of view. In the literature, several relevant examples of the use of metabolomics exist as a tool to improve chemical grouping by adding a biological perspective in *in vitro* studies, *in vivo* studies and in human exposure grouping.

#### 5.1.1. *In vitro* toxicological prediction

In a case study using human embryonic stem cells exposed to reference chemicals selected from the EPA's (Environmental Protection Agency) ToxCast™ chemical library,^103,104^ the authors observed that alterations in certain metabolic pathways (such as glutathione, arginine and proline metabolism, *etc*.) allowed them to construct a model with pharmaceutical agents of known developmental toxicity. The model was complemented with adverse outcomes from developmental toxicity studies (using ToxRefDB), among other sources of information. The prediction rate (accuracy of 73%) was comparable to that of animal studies.^104^ Similarly, the same authors presented another developmental toxicity prediction model (also in human embryonic stem cells) for teratogenic compounds.^105^ In this case, metabolomics alterations following such teratogenic exposure were used to train a model that was able to predict seven out of the eight other compounds with teratogenic capacity.

#### 5.1.2. *In vivo* toxicological prediction

One of the most complete works using metabolomics for predicting *in vivo* toxicology is a case study of phenoxy herbicides developed by van Ravenzwaay *et al.*^106^ In this study, the authors compared side by side a metabolomics-based approach for toxicology assessment to a classical approach. Rodents were exposed to different herbicides at several exposure levels and durations. Blood samples were analysed using a metabolomics workflow, and the metabolic patterns found to be dysregulated were linked to different organs affected by the studied herbicides following the OECD 407 repeated dose 28-day oral study guidelines.^106,107^ This readout was compared to a classical apical endpoint observation in animals. The authors clearly demonstrated that metabolomics could replace the classical experimental setup based on the good agreement between affected organs and NOAEL levels determined by both approaches.

In another study, the same authors studied the metabolomics alterations after exposure to 2-aminopropanol (a well-known chemical product with anti-cholinergic effects) and 3-aminopropanol (an analogue chemical but with data gaps to fulfil REACH requirements).^108^ The metabolomics approach unveiled similar metabolic patterns upon exposure to both compounds. Indeed, these metabolic alterations added an extra level of reliability to the study by proving the mechanistic similarity of both the new and the model substances using a read-across approach.^108^

### 5.2. Mechanism of toxicity

In the context of the adverse outcome pathway (AOP) framework, some studies have shown the potential use of metabolomics for understanding the mechanism of toxicity of chemicals.^10^ Utilizing scientific information from different levels of biological organization, AOPs represent a series of measurable key event (KE) and key event relationships (KER) that establish links between a molecular initiating event (MIE) and an adverse outcome (AO).^6,7,109^ Importantly, the AOP strategy aims to facilitate risk assessment evaluations by identifying AOs relevant to regulatory concerns.^6,8^ In this scenario, the role of the metabolomics approach is not only bringing new relevant scientific knowledge of already existing AOPs (*i.e.*, providing complementary biological information on the KE) but also contributing to identifying novel AOPs (by pointing out altered pathways and facilitating the prediction of a toxicological endpoint).^109^

#### 5.2.1. Identify and enlarge molecular KE

In a case study described by Davis *et al.*,^109^ the authors complemented the knowledge in an existing AOP by means of a metabolomics study. In this work, it was found that after 21 days of fathead minnow exposure to spironolactone, changes in endogenous metabolites (such as testosterone and other compounds) significantly correlated with changes in several reproductive endpoints.^109^ These findings provided potential molecular indicators of other biological perturbations that open the door to future AOP investigations and development.^109^ Similar studies^110,111^ also evaluated the importance of metabolomics for AOP development and risk assessment by studying the relationship between reproductive endpoints and metabolomic alterations.^109^ One of these studies demonstrated that earthworm metabolic profiles were correlated with their reproductive endpoints, with the advantage of a significant reduction in experimental time.^110^ Another example related changes in the earthworms’ metabolic profile and toxicological endpoints after exposure to three toxic compounds (CdCl_2_, atrazine and fluoranthene, representing three compound classes with different expected MOAs) at different concentration levels.^111^ Indeed, these metabolic alterations or fingerprints allowed to discriminate toxic mechanisms of action and show the relationship between the metabolic alterations and the toxicity outcome (*i.e.*, effect on reproduction).^111^

#### 5.2.2. Adverse outcome prediction

In the early 2000s, a study showed that after chronic exposure of *Daphnia magna* to eight toxicants, the metabolic profile (combined with other *omics* techniques) was able to predict future adverse effects on the growth, reproduction and survival of the organism.^112^ Ten years later, novel biomarkers that could predict the energetic physiological performance of marine mussels after exposure to copper and pentachlorophenol were evidenced by metabolomics.^113^ Similarly, in another study, metabolomic alterations were predictive of chronic impaired reproduction from acute metal exposure (copper and nickel) in *Daphnia pulex-pulicaria.*^114^ Moreover, Taylor *et al.*^115^ also described that metabolic biomarkers strongly predicted the reproductive impairment produced by cadmium, 2,4-dinitrophenol and propranolol to individual *Daphnia magna* in a 21-day OECD toxicity test regime.

#### 5.2.3. Identify different time of exposure

**Fig. 3:**
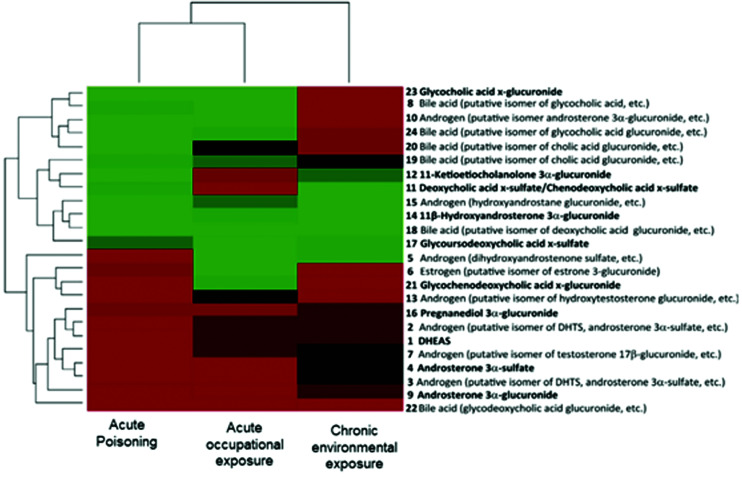
Bi-clustering analysis of the three types of dioxin exposure (acute poisoning, acute occupational exposure and chronic environmental exposure) based on the variations in the 24 steroid-related metabolite alterations. Compounds with increased levels are displayed in red, decreased compounds in green and compounds without changes in black. Each cohort is also compared with its own control. Reproduced from Jeanneret *et al.*^117^ with permission from Elsevier, copyright [2015].

A panel of 24 urinary steroid-related biomarkers (discovered in a prior untargeted metabolomics study^116^) was found to distinguish groups of individuals who had an episode of dioxin exposure from healthy controls.^117^ As shown in Fig. 3, many metabolites were significantly altered under three dioxin exposure conditions: (i) acute poisoning (Viktor Yushchenko case^118^), (ii) acute occupational exposure (Czech chemical workers intoxicated by dioxins in an herbicide production plant between 1965 and 1968) and (iii) chronic environmental exposure (neighbours and workers of municipal solid waste incinerators in France, where dioxins were present in the incineration fumes^119^). Overall, this study showed that a few endogenous metabolites (steroid-related compounds) could be used to distinguish different types of dioxin exposure with specific impacts on the urinary metabolome from control cases,^117^ emphasizing the relevant and dynamic information that metabolomics can provide about a toxicological state.

Overall, these selected examples supported the promising use of metabolomics for an improvement of the quality of the toxicological prediction. Metabolic profiling could thus enrich the characterization of substances and contribute to a more accurate toxicity assessment of the compound. Indeed, the biological information provided by metabolomics may contribute to a better description of the toxicological equivalence effects of the targeted substances.^28,79^

### 5.3. Point of departure

The point of departure (PoD) is commonly described as the dose that has no or a low (adverse) effect in a toxicological dose–response curve.^26,120^ Typically, the No-Observed-Adverse-Effect-Level (NOAEL) approach has been used to determine PoD in toxicological studies.^120^ However, during the 1980s, the benchmark dose (BMD) concept emerged, and it was established as the method of preference for the characterization of the point of departure for many health organizations world-wide.^120^ The possibility of deriving a benchmark dose from *omics* data was first explored by Thomas *et al.*^121^ using transcriptomic data (study of RNA transcripts) to identify doses at which individual cellular processes were altered.^26,121,122^

**Fig. 4:**
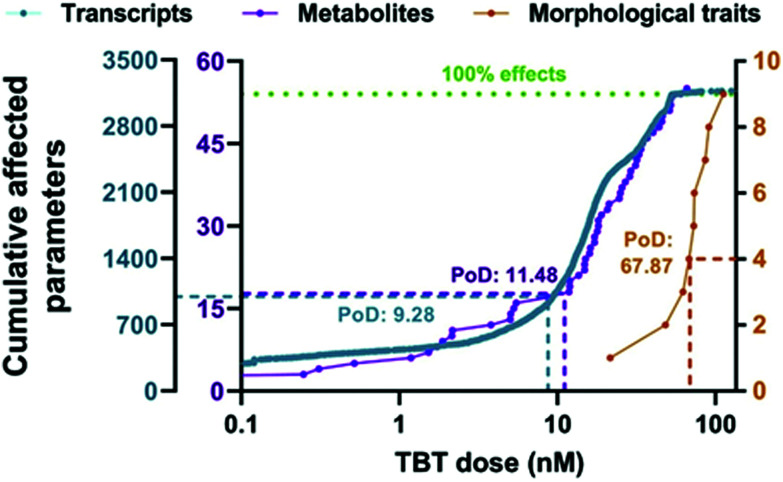
Accumulation plot of BMD values for the parameters of the different biological levels of zebrafish eleutheroembryos exposed to TBT: transcriptomics (in blue), metabolomics (in purple) and morphometrics (in orange). Points represent the number of parameters with a BMD value lower than a specific TBT concentration. The median BMDL values were used as PoD (point of departure) for the three biological levels and are interpolated in the graph as dotted lines. The green dotted line indicates 100% of the studied parameters for each. This figure is reproduced from Martínez R *et al.*^123^ with permission from Elsevier, copyright [2020].

A very recent case study showed the potential use of metabolomics for determining PoD in a BMD context.^123^ In this study, BMD analyses were performed to evaluate the dose–response effects of tributyltin (TBT) exposure (1.7–56 nM) on zebrafish eleutheroembryos. The results showed that the PoD obtained from the metabolomics changes (11.5 nM) was very similar to the PoD for transcriptomics (9.28 nM), and they were approximately one order of magnitude lower than the morphometric PoD (67.9 nM) or the median lethal concentration (LC_50_: 93.6 nM) (see Fig. 4),^123,124^ showing an improved sensitivity with regard to classical approaches. It has to be noted that an improved sensitivity does not automatically help regulators if the effect is transient and does not result in adversity. It might even lead to overly conservative regulation. Therefore, it is important to use this information in the context of a tiered approach where findings from metabolomics could be used to trigger additional investigations. With this study, the authors were able to separate the initially affected pathways (steroid biosynthesis metabolism) from those disrupted at higher concentrations (cell viability, general development, *etc*.)^123^ and eventually supported the use of metabolomics as early warning markers for toxicological studies. Furthermore, the inclusion of metabolomics for evaluating PoD could also prevent the lack of reproducibility or lack of linkage with the phenotype observed in some clinical studies.^123^

Other studies have compared the traditional determination of PoD based on a traditional dose–response strategy (such as the calculation of the NOAEL) with the metabolomics approach, and they found equivalent information.^125,126^ In addition, another study revealed that the variations in the metabolomics profile after hazardous chemical exposure were quantitatively linked to their toxicological endpoints.^127^ Therefore, the use of metabolomics for determining PoD could contribute to fast chemical screening for further toxicological studies.^26^

## 6. Metabolomics standardization for regulatory decision-making

Despite the current applications of metabolomics in the different domains of toxicology, its consideration in regulatory decision-making is in progress and under evaluation. Indeed, in 2016, a survey conducted by the European Centre for Ecotoxicology and Toxicology of Chemicals (ECETOC) showed that *omics* technologies were not yet extensively applied in the field of regulatory hazard assessment.^128,129^ Such a limited translation of metabolomics studies into reporting data for risk assessment was mainly attributed to the lack of well-defined best practices and reporting standards.^26^ Since then, several initiatives aimed at harmonizing metabolomics workflows and reporting in the field of regulatory toxicology have been started with the ultimate goal of better supporting decision-making about chemical compounds based on their mechanistic behaviour.^26,37^ An international expert group from multiple backgrounds supported by ECETOC designed the MERIT initiative (MEtabolomics standaRds Initiative in Toxicology). This initiative has recently published a series of best practices and reporting standards to facilitate the use of metabolomics in regulatory toxicology.^26,130^

These guidelines emphasize the relevance of adopting good practices throughout all the steps of the metabolomics workflows, from the experimental design to the management of data and metadata.^131,132^ With regard to the experimental design, this work highlights the importance of detailing test species, determining basal concentrations and using positive biological controls, along with the relevance of the design and the number of samples, the randomization and the management of batches. On the side of quality control and assurance, an extensive proposal of QC use (for system suitability, for intra-study, intra-lab or inter-lab comparison) is suggested. The sample collection and metabolite extraction are also covered in detail, with proposals for washing, quenching, extraction and injection. The guidelines also describe technique-specific conditions for the most common analytical strategies used in metabolomics (comprising NMR, LC-MS and GC-MS, either in untargeted or targeted mode). Finally, basic recommendations of data post-processing steps (covering topics related to targeted or untargeted acquisition strategies) and statistical analysis (with uni- and multivariate analysis) are provided to facilitate the interpretation of the results in terms of regulatory toxicology.^26^ Furthermore, the Organization for Economic Cooperation and Development (OECD) assembled a team of experts from industry, government agencies, regulators and academia to continue working on the Metabolomics Reporting Framework (MRF) Guidance Document to encourage the use of metabolomics in regulatory toxicology and to test and develop scientific outputs under the MERIT guidelines.

Although much younger than other *omics*, such as proteomics and genomics, metabolomics is now reaching a state of maturity in which well-established workflows and methodologies of demonstrated robustness are widely adopted by the community. Such consensus methods that are amenable to producing reliable results have the advantage of providing harmonized and trusted workflows; thus, they are ideal candidates to become the foundations of the progressive adoption of metabolomics in regulatory decision-making.

## 7. Conclusions

The need for toxicological evaluations of a growing number of compounds has highlighted metabolomics as a new methodological development in toxicology. Insights into the key concepts of metabolomics studies are provided in this review, including information on the experimental design, generation of biological samples, sample collection, stability and storage, sample treatment and sample analysis using different analytical platforms and acquisition strategies. Following an appropriate treatment of the obtained data, metabolomics can generate valuable biological information relevant for regulatory toxicology scenarios, such as in chemical grouping, benchmark dosing and understanding toxicity mechanisms. Overall, this review covers a number of key concepts that represent the basics of metabolomics vocabulary and highlight its broad potential for contributing to providing scientific knowledge and facilitating toxicity assessment and decision-making. Some examples found in the recent literature are given as guidance to the readers. Finally, some of the initiatives to harmonize metabolomics studies (following best practices) are cited since the reliability of metabolomics studies in the field of toxicology will strongly depend on a wide adoption and understanding of such working and reporting practices by both researchers in metabolomics and regulatory bodies.

## Conflicts of interest

Prof. Serge Rudaz belongs to the expert group “Metabolomics Reporting Framework (MRF)” from the Organization for Economic Cooperation and Development (OECD). The views expressed in this article are those of the authors and do not necessarily represent the views or policies of the OECD.
